# *In vitro* and *in vivo* *Areca catechu* crude aqueous extract as an anthelmintic against *Ascaridia galli* infection in chickens

**DOI:** 10.14202/vetworld.2019.877-882

**Published:** 2019-06-22

**Authors:** Wida Wahidah Mubarokah, Wisnu Nurcahyo, Joko Prastowo, Kurniasih Kurniasih

**Affiliations:** 1 Doctoral Program Parasitology Departement, Faculty of Veterinary Medicine, Universitas Gadjah Mada, Bulaksumur Sleman, Yogyakarta, 55281, Indonesia; 2 Department of Animal Health, Agriculture Extension College, The Polytechnique of Agricultural Development Yogyakarta Magelang, Jalan Magelang-Kopeng Km 7 Purwosari, Tegalrejo, Magelang, Jawa Tengah, 56192, Indonesia; 3 Department of Parasitology, Faculty of Veterinary Medicine, Universitas Gadjah Mada, Bulaksumur Sleman, Yogyakarta, 55281, Indonesia; 4 Department of Pathology, Faculty of Veterinary Medicine, Universitas Gadjah Mada, Bulaksumur Sleman, Yogyakarta, 55281, Indonesia

**Keywords:** *Areca catechu* crude aqueous extract, *Ascaridia galli*, chickens, *in vitro*, *in vivo*

## Abstract

**Aim:**

Intestinal parasites cause severe diseases at poultry farms, especially in developing countries, including Indonesia. Infections cause significant decreases in productivity at poultry farms. *Ascaridia galli* is a common nematode found in chickens with a prevalence of up to 92%. This study evaluates the *in vitro* and *in vivo* effects of *Areca catechu* crude aqueous extract (AAE) as an anthelmintic against *A. galli*.

**Materials and Methods:**

In the *in vitro* study, already-infected slaughtered chickens were collected from local slaughterhouses in Yogyakarta. The chicken intestines were carefully examined and transported to the Parasitological Laboratory of Gadjah Mada University, Yogyakarta. *A. galli* was collected from the intestines and put into a Petri dish containing 0.62% saline solution. Sixty *A. galli* individuals were soaked in different concentrations of the AAE for 3-6 h. The number of deceased *A. galli* was recorded by ascertaining whether they had ceased movement using a stereo microscope. The morphological characteristics of the parasites were studied. The *in vivo* study used 40 female chickens that were 1.5 months old and infected with the eggs of *A. galli*. Approximately 3000 infective eggs were found in each chicken. There were four treatment groups; Group A served as the control and was treated with Aquadestilata, Group B was treated with 26 mg/ml of AAE, Group C was treated with 79 mg/ml of AAE, and Group D was treated with 50 mg/ml of pyrantel pamoate. The AAE was applied using a pipette for 14 days. The observed parameters include the effect of the treatments on hematology, body weight (BW), and the number of *A. galli* eggs within each gram of fecal matter.

**Results:**

The AAE damaged the morphology, including the anterior end, posterior end, and vulva, of *A. galli in vitro*. The tegument of the anterior and posterior ends was slightly rougher, wrinkled, and damaged. The vulva and spicule ducts were irregular and indistinct. The average eggs per gram (EPG) in the untreated control increased *in vivo* from 1450±355.90 to 1975±325.96. The average EPG decreased from 1485±386.62 to 0±0.00 during 14 days of treatment of 79 mg/ml of AAE. The mean BW gain in the treated chickens was significantly (p<0.5) higher than the control. The treatment with 79 mg/ml of AAE increased the total erythrocyte count, total leukocyte count, hemoglobin, and packed cell volume values in the chickens.

**Conclusion:**

AAE can cause morphological changes and subsequent death in adult *A. galli* *in vitro* and effectively decrease ascariasis severity in chickens, therefore increasing overall BW *in vivo*.

## Introduction

Chicken meat and eggs are consumed in almost every country worldwide. Chicken meat is high in protein, and due to the price, is preferred more than beef in Indonesia [1]. Successful poultry production can be affected by many factors, including parasitic intestinal infections [2,3]. Intestinal parasites cause severe diseases at poultry farms, especially in developing countries, including Indonesia. The infection causes significant decreases in the productivity of poultry farms. *Ascaridia* *galli* is a common nematode found at high prevalence (92%) in chickens [4]. Heavily infected chickens may suffer from significant damage to the integrity of the intestinal villi [5,6]. Moreover, the intestinal mucosal defenses also change. One of said change is the thickening of the intestinal walls; the lumen itself appears to thicken as well due to enteritis. Another marked change is the appearance of the mucosal walls, which seems to be velvety. The aforementioned alterations lead to the nutrient absorption hindrance, resulting in slower body weight gain and a drop in egg production [6-9]. Many commercial anthelmintic drugs have been used in an attempt to control the parasitic infection of chickens.

Treatments using commercial anthelmintic drugs experience many problems, such as the high costs, environmental pollution risks, adverse effects on host health, and widespread anthelmintic resistance [10]. In addition, the drug residues in poultry meat can lead to carcinogenesis and resistance to causative agents. Drug resistance is caused by suboptimal therapeutic effects [3]. The development of the drug resistance of helminths to chemotherapeutical products deserves serious attention, and hence, it is necessary to find alternative treatments. Several studies have emphasized the importance of developing alternative ethnomedicinal agents extracted from plant materials such as *Areca catechu*. *A. catechu* (betel nut) belongs to the family *Arecaceae* and contains active substances such as flavonoids, tannins, saponins, monoterpenes, sesquiterpenes, phenols, quinones, and alkaloids (arecoline and arecaine) [11]. The seed of *A. catechu* is valuable as an anthelmintic because it contains alkaloids, arecolines, and tannins. Arecoline is toxic to some parasitic nematodes and can cause paralysis. Proanthocyanidin (tannin condensation) inhibits enzymes and degrades membranes, which in turn reduce the energy production of the parasite, ultimately causing death.

Therefore, in this study, we evaluated the *in vitro* and *in vivo* effects of *A. catechu* crude aqueous extract (AAE) on *A. galli*.

## Materials and Methods

### Ethical approval

All stages of the research were approved by the Ethical Committee of Gadjah Mada University, Indonesia with reference number 00126/04/LPPT/XI/2018.

### *In vitro* study

Already-slaughtered naturally infected chickens were collected from local slaughterhouses in Yogyakarta. The chicken intestines were carefully examined and transported to the Parasitological Laboratory of Gadjah Mada University, Yogyakarta. *A*. *galli* was obtained from the intestines and put into a Petri dish containing 0.62% saline solution. Sixty *A. galli* were soaked in each AEE concentration for 3-6 h. The number of deceased *A. galli* was recorded after no movement was observed from the individual under a stereo microscope. All image capturing processes were carried out using an Olympus Digital Camera under an Olympus CX21 microscope. The morphological characteristics of the parasites were identified.

AAE was prepared by cutting the *A. catechu* seeds into thin slices and drying them. Aquadestilata (100 ml) was then added to 10 g and 25 g of *A. catechu* to make up solutions of different concentrations. The solutions were then heated in an oven at 90°C for 15 min [12] then filtered to obtain AAE at concentrations of 100 mg/ml and 250 mg/ml.

### *In vivo* study

Forty females’ 1.5-month-old chickens were infected with the eggs of *A. galli*. Approximately 3000 infective eggs were found in each chicken. Fecal observation and examinations were conducted for 56 days, and the number of eggs in the feces was counted using the McMaster method. Treatments were applied to four different groups; Group A served as the control and was treated with Aquadestilata, Group B was treated with 26 mg/ml of AAE, Group C was treated with 79 mg/ml of AAE, and Group D was treated with 50 mg/ml of pyrantel pamoate. AAE was applied for 14 days using a pipette. The observed parameters include the effect of the treatments on hematology, body weight (BW), and the number of the *A. galli* eggs per gram (EPG).

Observation and examinations were conducted before the treatments (pre-treatment) and after the treatments (post-treatment). Blood samples (0.5 ml/chicken) were drawn from the brachialis vena (in wings) using a syringe. These samples were then placed into flasks containing EDTA. Hemoglobin (Hb) contents, packed cell volume (PCV), total erythrocyte counts (TEC), and total leukocyte counts (TLC) were examined [13].

### Statistical analysis

The quantitative data from the different treatments were statistically analyzed using one-way analysis of variance, t-test, and Chi-square analysis (x analysis). The morphology of the *A. galli* adult worms at different concentrations was determined using microscopic observations and analyzed descriptively.

## Results

**Figure-1:**
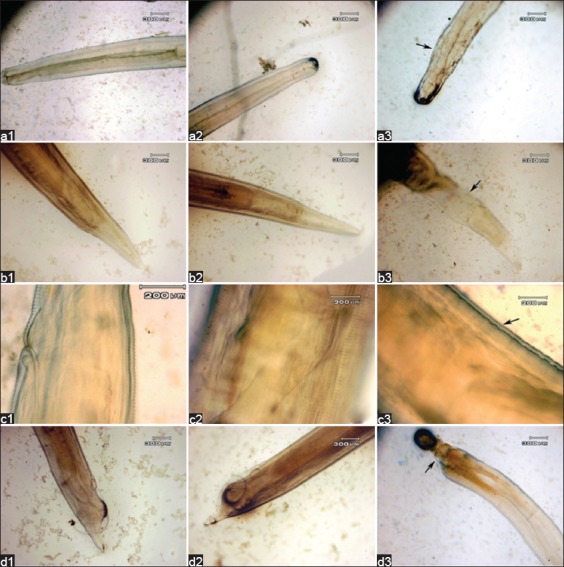
The morphology of *Ascaridia galli* not soaked in *Areca catechu* crude aqueous extract (AAE) (a1: Anterior end, b1: Female posterior end, c1: Vulva, d1: Male posterior end); the morphology of *A. galli* soaked in AAE at a concentration of 100 mg/ml (a2: Anterior end, b2: Female posterior end, c2: Vulva, d2: Male posterior end); and the morphology of *A. galli* soaked in AAE at a concentration of 250 mg/ml (a3: Anterior end, b3: Female posterior end, c3: Vulva, d3: Male posterior end).

The *in vitro* effects of AAE on the morphology of *A. galli* adult worms and the comparative characteristics are summarized in Figure-1. *A. galli* has a yellow-whitish elongated, cylindrical, and semi-transparent body that tapers toward both ends. The whole body is enclosed in a robust and pertinacious cover called a cuticle. The triangular mouth opens at the extreme anterior end. As shown in Figure-1, the teguments of the anterior ends that were exposed to AAE at concentrations of 250 mg/ml (a3) and 100 mg/ml (a2) were found to be slightly rougher and more wrinkled than those of the control (a1). The teguments of the posterior ends of females at concentrations of 250 mg/ml (b3) and 100 mg/ml (b2) were more damaged and wrinkled than the control (b1). The vulva appears to be irregular at concentrations of 250 mg/ml (c3) and 100 mg/ml (c2), while the indistinct duct of the vulva is observed, compared to the control (c1). The posterior end of the male appears to be irregular at concentrations of 250 mg/ml (d3) and 100 mg/ml (d2), while the indistinct line of the spicule forms is observed, compared to the control (d1).

The results of the variance analysis show that the application of AAE has a significant effect on the mortality rates of adult *A. galli* after 6 h of treatment (p<0.05). The soaking durations at concentrations of 150 mg/ml to 250 mg/ml are significantly different (p<0.05). The death of *A. galli* adults occur at a dosage of 125 mg/ml after 6 h of treatment. The most effective concentration for killing adult *A. galli* was found to be 250 mg/ml, which is equivalent to the chemical anthelmintics of pyrantel pamoate (Table-1).

**Table-1:** The effects of AAE concentrations on the mortality rates of *Ascaridia galli* after immersion for 3-6 h.

Treatments	3 h	6 h
NaCl 0.62%	0.00±0.00^a,g^	0.00±0.00^a,g^
AAE 100 mg/ml	0.00±0.00^a,g^	0.00±0.00^a,g^
AAE 125 mg/ml	0.00±0.00^a,g^	10.00±0.00^b,h^
AAE 150 mg/ml	0.00±0.00^a,g^	11.70±0.00^b,h^
AAE 175 mg/ml	0.00±0.00^a,g^	11.70±0.00^b,h^
AAE 200 mg/ml	0.00±0.00^a,g^	33.00±0.00^c,h^
AAE 225 mg/ml	1.70±0.107^a,g^	48.30±0.10^d,h^
AAE 250 mg/ml	5±0.00^b,g^	95.00±0.00^e,h^
Pyrantel pamoat 5%	8.3±0.10^c,g^	91.70±0.00^e,h^

a,b,c,d,e,f Different superscripts in a column indicate significant differences (p<0.05).

g,h Different superscripts in a row indicate significant differences (p<0.05), AAE=*Areca catechu* crude aqueous extract

The results of the variance analysis, summarized in Table-2, show that treatment with 79 mg/ml of AAE caused a significant difference (p<0.05) compared to the control, while treatment with 26 mg/ml of AAE did not cause any significant difference. Treatment with 79 mg/ml of AAE caused a 90.06% decrease in EPG. This result is similar to that of the treatment with 50 mg/ml of the anthelmintic pyrantel pamoate (89.93%). The most effective dosage of AAE to decrease EPG is 79 mg/ml.

**Table-2:** The effect of AAE on fecal egg counts.

Treatment	Pre-treatment	Post-treatment day 7	Post-treatment day 14	Mean FECR (%)
Aquadestilata	1450±355.90	1655±386.90	1975±325.96	−16.73^a^
AAE 26 mg/ml	1285±434.01	1355±405.14	1555±238.57	−10.11^a^
AAE 79 mg/ml	1485±386.62	295±149.91	0±0.00	90.06^b^
Pyrantel pamoate 50 mg/ml	1415±539.57	285±120.30	0±0.00	89.93^b^

a,b,c Different superscripts indicate significant differences (p<0.05). AAE=*Areca catechu* crude aqueous extract, FECR=Fecal egg count reduction

The t-test analysis in Table-3 shows that there is a significant disparity in the weekly weight gain between the control chicken and those treated with different dosage of AAE. There is also a significant difference (p<0.05) among pyrantel pamoate and the treatments of 26 mg/ml and 79 mg/ml of AAE. The application of 79 mg/ml of AAE results in the most significant BW gain, though it is not equal to the efficacy of pyrantel pamoate.

**Table-3:** The effect of AAE on chicken body weight (g).

Treatment	Pre-treatment	Post-treatment day 7	Post-treatment day 14	Mean weight gain
Aquadestilata	1132±77.29	1130±76.16	1124±71.06	−6±15.10^a^
AAE 26 mg/ml	1130±66.16	1132±66.80	1134±66.03	2±0.77^b^
AAE 79 mg/ml	1140±78.32	1186±80.58	1268±88.04	82±7.46^c^
Pyrantel pamoate 50 mg/ml	1142±46.62	1192±41.31	1280±37.71	88±3.60^d^

a,b,c Different superscripts indicate significant differences (p<0.05). AAE=*Areca catechu* crude aqueous extract

Table-4 shows that there is a significant difference in TEC and TLC (p<0.05) between pre-treatment and post-treatment for all treatments. There is also a considerable difference in the post-treatment (p<0.05) between the application of AAE and the control. The most significant effect of 79 mg/ml of AAE exceeds the number of TEC as compared to the efficacy of pyrantel pamoate, but it has not been able to exceed the amount of TLC as compared to the efficacy of pyrantel pamoate. There is no significant difference in Hb and PCV between pre-treatment and post-treatment, but there is a substantial difference in Hb between AAE and the control. The most significant effect was found from 79 mg/ml of AAE, which is similar to the efficacy of pyrantel pamoate.

**Table-4:** The effects of AAE on chicken hematology.

Blood parameters	Group	Pre-treatment	Post-treatment
Hb (g/dl)	Aquadestilata	9.95±1.26^a,f^	8.60±0.63^b,f^
	AAE 26 mg/ml	10.08±0.89^a,f^	9.07±1.23^b,g^
	AAE 79 mg/ml	9.45±1.25^a,f^	10.32±0.71^b,g^
	Pyrantel pamoate 50 mg/ml	9.50±0.97^a,f^	10.02±0.75^b,g^
PCV (%)	Aquadestilata	31.58±2.50^a^	30.83±1.63^a,f^
	AAE 26 mg/ml	31.50±1.07^a,f^	30.58±1.075^a,f^
	AAE 79 mg/ml	31±3.35^a,f^	31.50±1.38^a,f^
	Pyrantel pamoate 50 mg/ml	30.67±2.66^a,f^	31.33±1.37^a,f^
TLC (10^3^/mm^3^)	Aquadestilata	34.75±7.67^a,f^	30.08±1.63^b,f^
	AAE 26 mg/ml	34.75±5.95^a,f^	29.33±9.84^b,f^
	AAE 79 mg/ml	20.42±4.05^a,g^	21.42±7.45^a,g^
	Pyrantel pamoate 50 mg/ml	17.67±2.42^a,g^	22.58±3.41^a,g^
TEC (million/UI)	Aquadestilata	3.17±0.93^a,f^	3.00±0.34^a,f^
	AAE 26 mg/ml	2.82±0.25^a,g^	2.70±0.33^a,g^
	AAE 79 mg/ml	2.73±0.35^a,g^	3.10±0.22^b,g^
	Pyrantel pamoate 50 mg/ml	2.97±0.34^a,g^	3.07±0.23^a,g^

a,b Different superscripts in a row indicate significant differences (p<0.05).

f,g Different superscripts in a column indicate significant differences (p<0.05). AAE=*Areca catechu* crude aqueous extract, Hb=Hemoglobin, PCV=Packed cell volume, TLC=Total leukocyte count, TEC=Total erythrocyte count

## Discussion

The phytochemical screening of *A. catechu* shows that it contains flavonoids, tannins, saponins, monoterpenes, sesquiterpenes, phenols, quinones, and alkaloids (arecoline and arecaine) [11]. The death of the adult *A. galli* may be due to the presence of tannin and saponin in *A. catechu*. Tannin can affect the adult *A. galli* both directly and indirectly. Tannin affects *A. galli* directly by attaching to its cuticle [14], which plays an essential multi-functional role in the protective and selective absorption functions of *A. galli*.

Furthermore, the cuticle of the nematode becomes a primary target site for anthelmintic drugs [15]. These findings are similar to those of Roy *et al*. [16], whereby *Acacia* *oxyphylla* induces cuticular aberrations in regular striations and damages the epicuticle and cuticle of the *A. galli* adult worms. Another study reports that *Calendula micrantha* extract wrinkles the surface and causes the loss of striations along the cuticle of *A*. *galli* [17]. The active compound isolated from *A. oxyphylla* causes the disorganization of the body surface of *A. galli* [16].

Similar results also show that tannin can damage the cuticle of *Haemonchus contortus*. Sambodo *et al*. [18] evaluated the cuticle change of *H. contortus* with longitudinal and transversal wrinkles after *in vitro* exposure to *Biophytum persianum*, which is rich in condensed tannin. Wrinkles in the cuticle and anterior end of *H. contortus* were observed by Martínez-Ortíz-de-Montellano *et al*. [19]. The cuticle reveals the form of adult worms and is involved in its motility and exchanges with the environment, including metabolic exchanges with the digestive tract of its host [19]. In addition, a decrease in body length and width caused by tannin occurs in *H. contortus* [20].

Phytochemical constituents that act to combat parasites utilize different mechanisms. Lorent *et al*. [21] found that the cytotoxic activity of saponins means they can form pores on cell membranes which disrupt the ionic balance of the cell, resulting in cell lysis and death. Nyambuya *et al*. [22] found that the alkaloid extracted from *Combretum zeyheri* can inhibit transport across cell membranes. In general, the mechanisms of anthelmintic activity in plant-based extracts may interfere with and combine cell membranes to elicit changes to cell composition. Consequently, this activity induces membrane destabilization, changes in membrane permeability, and the loss of membrane potentials, causing cell lysis, which subsequently damages the cuticle and causes morphological failure.

The results of the EPG in this study are consistent with a study by Feroza *et al*. [23], which shows that the application of the ethanolic extract of papaya (*Carica papaya*) and neem (*Azadirachta indica*) seeds can decrease EPG values (by 99.5% and 97.7%, respectively). In addition, Akter *et al*. [24] suggest that the application of pineapple leaf extract decreases the average EPG of *A. galli* from 300±11.07 to 60±7.40 within 28 days of pineapple treatment. This observation is consistent with the findings of Begum *et al*. [25] who have conducted an *in vivo* study of aqueous pineapple leaf extract and reported a decrease in the EPG count of *A. galli* after 21 days post-treatment. Other studies show that different doses of pineapple (*Ananas comosus*) peel juice (PPJ) at 504 mg/kg BW PPJ and 255 mg/kg BW PPJ are considered effective on day 14 with 91.1% and 93.6% fecal egg count reductions test (%) of *A. galli*, respectively. Cormanes *et al*. [26] showed that the application of flubendazole (1.43 mg/kg) reduces the egg numbers of *A. galli* by 65% and 88% on day 3 of treatment and by 99% and 97% on day 35 post-treatment. Tarbiat *et al*. [27] suggested that a dose of 5000 mg/kg of *Artemisia* *campestris* essential oil shows a high nematocidal activity (72.1% FECR) in *H*. *contortus* on day 7 post-treatment [28]. In this study, the decrease in EPG value may be caused by the presence of tannin and saponin in *A. catechu*. The alkaloids, flavonoids, saponins, steroids, anthraquinones, and tannins extracted from plants using hot water extraction and methanol can hamper embryonization and the egg hatching process causing the death of larvae [29].

The BW changes in this study are consistent with those of Khokon *et al*. [30], which suggests that the application of 1 g/kg of neem leaves causes a significant increase in chicken BW in the treatment group compared to the control. Raza *et al*. [31] showed that the application of *A. comosus* extracts also increases chicken BW. According to Akter *et al*. [24], the increase in chicken BW due to the use of pineapple leaf extract is significantly higher than that in control (p<0.01). BW increases when the number of parasitic nematodes decreases by allowing increased absorption of nutrients into the chicken’s body.

The blood examination (TEC, Hb, and PCV) results at 79 mg/ml AAE concentration are consistent with those of Akter *et al*. [24] suggesting that pineapple leaf extract increases chicken TEC, Hb, and PCV values and decreases the TLC value. A study by Khokon *et al*. [30] observes an increase in chicken TEC, Hb, and PVC values after the application of neem leaves (1 g/kg of BW). Therefore, the difference in TLC values in this study may result from the presence of other microorganisms that infected the chickens during the induction phase.

## Conclusion

AAE can cause changes in morphology and the death of *A. galli* adult parasitic nematodes *in vitro*. AAE can also effectively decrease ascariasis severity and increase BW in chickens.

## Authors’ Contributions

KK designed, managed, and supervised the study. WWM collected and analyzed samples. WN, WWM, JP, and KK arranged, analyzed, and wrote the manuscript. JP organized all observations in the experiment and wrote the manuscript. All authors have read and approved the final manuscript.

## References

[1] Henuk Y.L (2015). The role of family poultry production systems in developing countries. Kaen Kaset.

[2] Nnadi P.A, George S.O (2010). A cross-sectional survey on parasites of chickens in selected villages in the subhumid zones of South-Eastern Nigeria. J. Parasitol. Res.

[3] Ozaraga B, Sylvia M.A, Ozaraga I, Barrios M.B (2015). Ethnobotanical dewormer composition for free-range native chickens. Mindanao J. Sci. Technol.

[4] Offionga E.E.A, Obiokub O.E, Umohb J.U, Essienc C.A, Idiongc N.B (2013). A survey of gastrointestinal helminths of local chickens in Abak local government area of Akwa Ibom State. Int. J. Sci.

[5] Alrubaie A.L (2015). Effect of alcoholic extract of *Curcuma longa* on *Ascaridia* infestation affecting chicken. Indian J. Exp. Biol.

[6] Salam S.T (2015). Ascariasis in backyard chicken-prevalence, pathology and control. Int. J. Recent Sci. Res.

[7] Das G, Kaufmann F, Abel H, Gauly M (2010). Effect of extra dietary lysine in *Ascaridia galli* infected grower layers. Vet. Parasitol.

[8] Rivera K.C, Cortes J.A.V, Lara A.A, Grandvallet L.A.L (2016). A diagnostic survey of gastroenteric helminths in backyard poultry of a rural village in Mexican tropics. J. Agric. Biol. Sci.

[9] Teixeira M, Monteiro J.P, Catenacci L.S, Rodrigues M.L.A, Sato M.C.B (2012). Ascaridiasis in peafowl *Pavo cristatus* (*Phasianidae*) due to *Ascaridia galli* Schrank, 1788. J. Zoo Wildl. Med.

[10] Vercruysse J, Albonico M, Behnke J.M, Kotze A.C, Prichard R.K, McCarthy J.S, Montresor A, Levecke B (2011). Is anthelmintic resistance a concern for the control of human soil-transmitted helminths. Int. J. Parasitol. Drugs Drug Resist.

[11] Amudhan M.S, Begum V.H, Hebbar K.B (2012). A review on phytochemical and pharmacological potential of *Areca catechu* L. seed. Int. J. Pharm. Sci. Res.

[12] Widiarso B.P, Kurniasih K, Prastowo J, Nurcahyo W (2018). Morphology and morphometry of *Haemonchus contortus* exposed to *Gigantochloa apus* crude aqueous extract. Vet. World.

[13] Qamar M.F, Maqbool A (2012). Biochemical studies and serodiagnosis of haemonchosis in sheep and goats. J. Anim. Plant. Sci.

[14] Zhong R.Z, Sun H.X, Liu H.W, Zhou D.W (2014). Effects of tannin acid on *Haemonchus contortus* larvae viability and immune responses of sheep white blood cells *in vitro*. Parasite Immunol.

[15] Alvarez L.I, Mottier M.L, Lanusse C.E (2007). Drug transfer into target helminths parasites. Trends Parasitol.

[16] Roy B, Dasgupta S, Manivel V, Parameswaran P.S, Giri B.R (2012). Surface topographical and ultrastructural alterations of *Raillietina echinobothrida* and *Ascaridia galli* induced by a compound isolated from *Acacia oxyphylla*. Vet. Parasitol.

[17] Hassanain M.A, Rahman E.H.A, Khalil F.A.M (2009). New scanning electron microscopy look of *Ascaridia galli*(Schrank 1788) adult worm and its biological control. Res. J. Parasitol.

[18] Sambodo P, Prastowo J, Kurniasih K, Indarjulianto S (2018). *In vitro* potential anthelmintic activity of *Biophytum petersianum* on *Haemonchus contortus*. Vet. World.

[19] Martínez-Ortíz-de-Montellano C, Arroyo-López C, Fourquaux I, Torres-Acosta J.F.J, Sandoval-Castro C.A, Hoste H (2013). Scanning electron microscopy of *Haemonchus contortus* exposed to tannin-rich plants under *in vivo* and *in vitro* conditions. Exp. Parasitol.

[20] Kuchai J.A, Ahmad F, Chishti M.Z, Tak H, Ahmad J.A.S, Razool M (2012). A study on morphology and morphometry of *Haemonchus contortus*. Pak. J. Zool.

[21] Lorent J.H, Quetin L.J, Mingeot L.M.P (2014). The amphiphilic nature of saponins and their effects on artificial and biological membranes and potential consequences for red blood and cancer cells. Org. Biomol. Chem.

[22] Nyambuya T, Mautsa R, Mukanganyama S (2017). Alkaloid extracts from *Combretum zeyheri* inhibit the growth of *Mycobacterium smegmatis*. BMC Complement. Altern. Med.

[23] Feroza S, Arijo A.G, Zahid I.R (2017). Effect of papaya and neem seeds on *Ascaridia galli* infection in broiler chicken. Pak. J. Nematol.

[24] Akter M.K, Jalal U, Maksudur R, Fahima B.A, Bazlar R, Mahmudul H (2016). Studies on the prevalence of ascariasis in indigenous chickens in Gaibandha District and treatment by pineapple leaves extract. Res. Agric. Livest. Fish.

[25] Begum S, Mostofa M, Alam A.K.M, Hossain R, Barman M.F, Ali A.A.M, Mian R (2010). Comparative efficacy of leaves extract of neem and bishkatali with patent drugs piperazine and levamisole against ascariasis of indigenous chicken. Int. J. Biol. Res.

[26] Cormanes J.M.Y, Harvie P.P, Ana M.M.Q (2016). *In vivo* anthelmintic activity of pineapple (*Ananas comosus* Merr.) fruit peeling juice in semi-scavenging Philippine native chicken naturally co-infected with *Ascaridia galli* and *Heterakis gallinarum*. Livest. Res. Rural Dev.

[27] Tarbiat B, Jansson D.S, Moreno L, Lanusse C, Nylund M, Tydén E, Höglund J (2016). The efficacy of flubendazole against different developmental stages of the poultry roundworm *Ascaridia galli* in laying hens. Vet. Parasitol.

[28] Abidi A, Sebai E, Dhibi M, Alimi D, Rekik M, B'chir F, Maizels R.M, Akkari H (2018). Chemical analyses and anthelmintic effects of *Artemisia campestris* essential oil. Vet. Parasitol.

[29] Eguale T, Tadesse D, Giday M (2011). *In vitro* anthelmintic activity of crude extract of five medicinal plants against egg hatching and larval development of *Haemonchus contortus*. J. Ethnopharmacol.

[30] Khokon J.U, Sharif U.Z, Emran H.S, Mohammad A.R, James J.K, Mahbub M (2014). Efficacy of neem leaf extract against ascariasis in indigenous chicken. Int. J. Nat. Soc. Sci.

[31] Raza A, Muhammad F, Bashir S, Aslam B, Anwar M.I, Naseer M.U (2016). *In vitro* and *in vivo* anthelmintic potential of different medicinal plants against *Ascaridia galli* infection in poultry birds. World. Poult. Sci. J.

